# Two mutations G335D and Q343R within the amyloidogenic core region of TDP-43 influence its aggregation and inclusion formation

**DOI:** 10.1038/srep23928

**Published:** 2016-03-31

**Authors:** Lei-Lei Jiang, Jian Zhao, Xiao-Fang Yin, Wen-Tian He, Hui Yang, Mei-Xia Che, Hong-Yu Hu

**Affiliations:** 1 State Key Laboratory of Molecular Biology, Institute of Biochemistry and Cell Biology, Shanghai Institutes for Biological Sciences, Chinese Academy of Sciences. 320 Yue-Yang Road, Shanghai 200031, China

## Abstract

TDP-43 is a DNA/RNA binding protein associated with TDP-43 proteinopathies. Many mutations have been identified in the flexible C-terminal region, which is implicated in the disease pathology. We investigated four point mutations in the amyloidogenic core region (residues 311–360) of TDP-43 by biochemical and spectroscopic methods. We found that the G335D mutation enhances the aggregation and inclusion formation of TDP-43 and this mutant in TDP-35 (the C-terminal fragment of 35 kDa) exaggerates the antagonist effect on RNA processing by endogenous TDP-43; whereas Q343R gives an opposite effect. As a comparison, M337V and Q331K have very little impact on the aggregation and inclusion formation of TDP-43 or TDP-35. NMR structural analysis showed that the G335D mutant in the core region forms a loop linker between the two α-helices and promotes α-to-β transition, but Q343R loses the second helix and consequently the structural transformation. Thus, the propensity of structural transformation in the amyloidogenic core of TDP-43 determines its aggregation and inclusion formation. This study may provide a molecular mechanism of the TDP-43 proteinopathies caused by genetic mutations.

TDP-43 (TAR DNA-binding protein of 43 kDa) is a DNA/RNA binding protein containing two RNA recognition motifs (RRMs) that participate in RNA binding and a long C-terminal glycine-rich region (GRR) that is involved in protein-protein interactions^1^,^2^. Also, a nuclear localization signal (NLS) sequence between the N- terminal and the RRM domains is important for interacting with the nuclear transport factors^3^. Previous studies have revealed that ubiquitination, mislocalization, fragmentation and aggregation of TDP-43 in cytoplasm are clinically associated with amyotrophic lateral sclerosis (ALS) and frontotemporal lobar degeneration (FTLD) diseases^4^,^5^. The pathological hallmark of these neurodegenerative diseases is the formation of TDP-43 aggregates or inclusions in neuronal cells, as called TDP-43 proteinopathies^2^,^6^,^7^.

TDP-43 is mainly localized in nucleus and plays multiple roles in transcriptional repression, pre-mRNA splicing, translational regulation and stability^8^,^9^,^10^,^11^. However, it may experience redistribution from nucleus to cytoplasm, form aggregates or inclusions in cytoplasm and lose its normal function in clinical observations^12^,^13^,^14^. A wealth of studies have revealed that mislocalization and aggregation of the C-terminal fragments of TDP-43 including TDP-35 (~35 kDa) and TDP-25 (~25 kDa) are critical for TDP-43 proteinopathies^5^,^15^,^16^, and especially the C-terminal GRR domain is of great importance for TDP-43 aggregation^17^,^18^,^19^,^20^. Some studies suggest that the Gln/Asn-rich domain in the C terminus is aggregation-prone^21^,^22^,^23^. In addition, different amyloidogenic cores for TDP-43 aggregation have been defined in the C-terminal region, including sequences 286–331^24^, 311–360^25^ and 342–366^26^. The GRR domain of TDP-43 is also responsible for the reversible, dynamic protein inclusions with other RNA-binding proteins, which confer the ability to consolidate RNA transcripts into the ribonucleoprotein (RNP) granules^27^,^28^,^29^.

More than 40 mutations in TDP-43, mainly in the C-terminal GRR domain, have been identified in familial and sporadic cases of ALS and FTLD^7^,^23^,^30^. Among these mutations, A315T is the most concrete one that can enhance TDP-43 aggregation and inclusion formation, impair axonal transportation of mRNA, reduce RNA granule density and mobility, and cause neurotoxicity^24^,^27^,^31^,^32^. As known, mutations in TDP-43 may influence its aggregation, dysfunction, axonal transportation and even RNA granule formation, implying the possible amyloidogenic and neurotoxic properties of TDP-43 mutants in ALS and FTLD. Therefore, studies of individual mutations in TDP-43 are beneficial to elucidating the TDP-43 proteinopathies.

Previously, we identified an amyloidogenic core (residues 311–360) in the C-terminal flexible region of TDP-43^25^. By using various biochemical and biophysical techniques, we revealed that the core is prone to structural transformation from an α-helix to a β-sheet when its aggregation occurs, and consequently triggers TDP-43 aggregation and cytoplasmic inclusion formation. In this study, we focused on the genetically-related mutations within the amyloidogenic core of TDP-43. We found that two mutants, G335D and Q343R, exert significant effects on the aggregation and inclusion formation of TDP-43 as well as TDP-35. Structural information of the mutant amyloidogenic-core fragments was obtained, and the importance of the mutants in the aggregation and inclusion formation of TDP-43 was also confirmed *in vitro* and in cells. Our findings implicate pathological impact of the mutations on the inclusion formation and dysfunction of TDP-43, and bring light to the pathogenesis of the related neurodegenerative diseases.

## Results

### Two mutations G335D and Q343R affect TDP-43 aggregation

**Figure 1:**
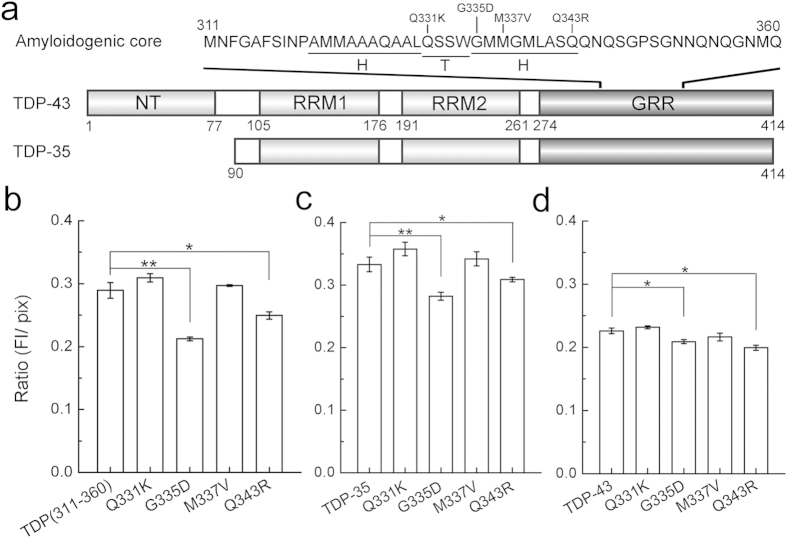
Assaying the aggregation abilities of TDP-43 variants by GFP-fusion method. (**a**) Schematic representation of the domains in TDP-43 and the genetic mutations in the amyloidogenic core. NT, N-terminal domain; RRM, RNA recognition motif; GRR, glycine-rich region, residues 274–414. TDP-35, the C-terminal fragment of TDP-43, residues 90–414; amyloidogenic core, residues 311–360. H, α-helix; T, turn. (**b**) The FI/pix ratios of TDP(311–360) and its mutants. FI/pix denotes the ratio of fluorescence intensity to protein amount. The relative GFP-fused protein amount was quantified by recording the grayscale (pixel) of a band in the gel. (**c**) The FI/pix ratios of TDP-35 and its mutants. (**d**) The FI/pix ratios of full-length TDP-43 and its mutants. All data from three independent experiments were analyzed statistically by one-way ANOVA and represented as Mean ± SD (n = 3). *p < 0.05; **p < 0.01.

As preciously described, many mutations in the C-terminal GRR domain of TDP-43, especially in its amyloidogenic core, have been identified in familial and sporadic cases of ALS and FTLD^7^,^33^. So, we focused on four mutations (Q331K, G335D, M337V and Q343R) within the secondary structure region of the amyloidogenic core (Fig. 1a). To assess the effects of these mutants on the aggregation, we prepared a series of GFP-tagged constructs at their C termini and overexpressed them in *E. coli* to report the aggregation properties of the fusion proteins. As our previous report^25^, the ratio of the fluorescence intensity to the protein amount (FI/pix) represents the aggregation ability of a GFP-fused fragment. Compared with the wild-type core fragment (TDP(311–360)), the G335D mutation in this fragment significantly enhanced the aggregation propensity, while Q343R gave a slight increase in its aggregation (Fig. 1b). However, the Q331K and M337V mutants did not exhibit any obvious effect on the aggregate formation. This observation was also verified by the mutations in TDP-35 (Fig. 1c) and full-length TDP-43 (Fig. 1d). These results imply that two mutants G335D and Q343R have altered their aggregation properties.

### Effects of the mutations on aggregation of the amyloidogenic core

**Figure 2:**
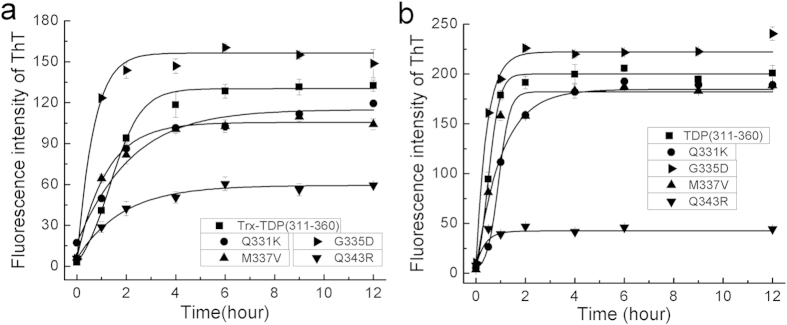
Aggregation of TDP(311–360) and its mutants *in vitro*. (**a**) Time courses showing aggregation of the Trx fusions of TDP(311–360) and its mutants upon thrombin cleavage. The aggregation ability was represented by ThT fluorescence intensity. The protein concentration was 100 μM. Trx, thioredoxin. (**b**) Time courses showing aggregation of the purified TDP(311–360) and its mutants. The peptide concentration was 100 μM. Shown are three independent experiments (n = 3).

To further investigate the impact of the mutations on TDP-43 aggregation, we compared their aggregation properties *in vitro* by ThT assay. Firstly, we analyzed time courses of the core fragment and its mutants fused with thioredoxin (Trx-TDP(311–360)) upon thrombin cleavage (Fig. 2a). As a result, the G335D mutant caused a higher fluorescence enhancement of the ThT dye, suggesting that this mutant forms amyloidogenic aggregates more readily than the wild-type TDP(311–360). However, the Q343R mutant gave rather low fluorescence enhancement during incubation, indicating that it cannot form amyloidogenic aggregates fully. Both Q331K and M337V mutants exhibited fluorescence enhancement abilities similar to the wild type, although these two mutants in TDP-43 were reported to cause alteration of RNA splicing^34^. Then, we purified these peptide fragments and measured their aggregation abilities by ThT assay. The data reconfirmed that the G335D mutant was more prone to aggregation and Q343R significantly reduced the aggregation ability (Fig. 2b), whereas Q331K and M337V had similar abilities to form aggregates. Of note, we also observed visible particle matters for Q343R after incubation. Although the possibility that the mutation may alter their structures and ThT binding abilities could not be excluded, we think ThT binding may reflect the aggregation properties of peptides or proteins as previous studies indicated^35^. Taken together, these results demonstrate that the mutations G335D and Q343R significantly alter the aggregation properties of the amyloidogenic core fragment.

### Solution structures of the G335D and Q343R mutants of the amyloidogenic core fragments

**Figure 3:**
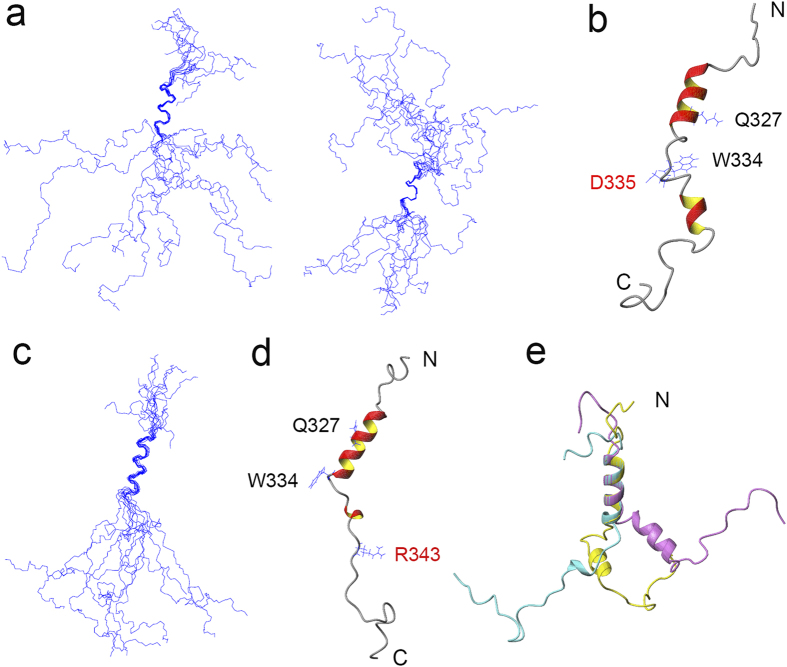
NMR solution structures of the G335D and Q343R mutants of TDP(311–360) peptide. (**a**) Ensemble of the 10 lowest-energy structures of G335D superimposed on backbones of the first helix (left) or the second helix (right), respectively. (**b**) Ribbon diagram of a representative structure of G335D showing a helix-loop-helix structure. (**c**) Superposition of the backbone traces of the 10 lowest-energy structures of Q343R. (**d**) Ribbon representation of the backbone structure of Q343R showing a helix and a flexible tail. (**e**) Overlay of the structures of TDP(311–360) (purple), G335D (yellow) and Q343R (cyan). The N-terminal α-helix was selected to overlap. N, N terminus; C, C terminus. The structures are displayed with MOLMOL.

We previously demonstrated that the amyloidogenic core fragment of TDP-43 (TDP(311–360)) forms a helix-turn-helix structure (Fig. 1a)^25^. The two helices are from residues 321 to 330 and 335 to 343 respectively, the turn is in between the two helices, and several NOEs are observed between Gln327 and Trp334. To compare the structures of the amyloidogenic core mutants with that of the wild type, we solved the solution structures of the G335D and Q343R peptides in solution by using NMR method as described previously^25^. The experimental restraints and structural statistics for the G335D and Q343R mutants of TDP(311–360) are displayed in Supplementary Table S1. Compared with the wild type, the G335D mutant still exhibited two α-helices from residues 321 to 330 and 339 to 343 (Fig. 3a,b), but different from the wild type that forms a turn between the helices. The flexible linker region in G335D extended from residue 331 to 338. There were no long-range NOEs observed between the two helices; hence, the orientation of the two helices was less restricted and the overall structure was more extended (Fig. 3a). On the other hand, Q343R exhibited only one α-helix from residue 322 to 333, and the long-range NOEs between Gln327 and Trp334 had not been detected (Fig. 3c,d). Thus, in comparison with the wild type (Fig. 3e), the structure of G335D is composed mainly of a helix-loop-helix structure. It is speculative that extension of the flexible loop linker in the G335D mutant makes it more readily transform from helix-loop-helix to a hairpin-like β-sheet structure, which may enlarge the possibility of forming aggregates or amyloid. However, due to loss of the second α-helix, Q343R may lose its ability to transform into a hairpin-like β-sheet structure and consequently to form the β-sheet-rich aggregates either.

### Structural transformation of the amyloidogenic core mutants

**Figure 4:**
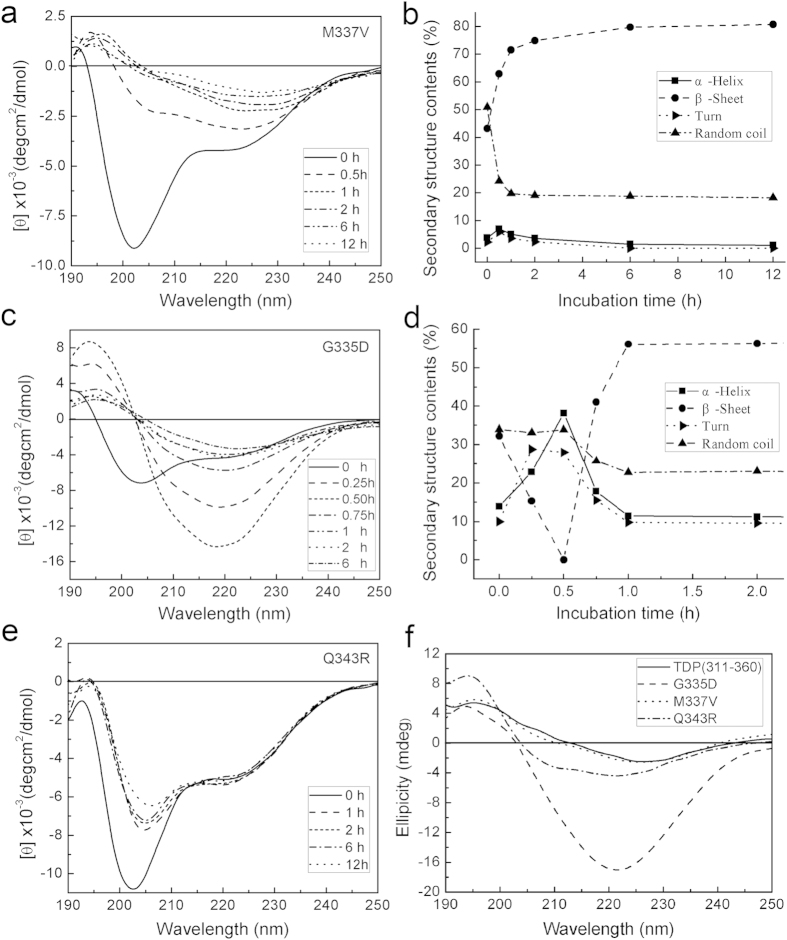
Structural transformation and aggregation of the TDP(311–360) mutants. (**a,c,e**) Time courses of the CD spectrum changes of M337V (**a**), G335D (**c**) and Q343R (**e**) in solution during incubation processes. For time-course incubation, the concentration of each protein was 100 μM in a buffer (100 mM phosphate, 100 mM NaCl, pH7.0). (**b, d**) Secondary-structure content changes of M337V (**b**) and G335D (**d**) at various incubation times. The data were obtained from analyzing the CD spectra (**a**,**c**) with a computer program. (**f**) Comparison of the solid-state CD spectra of TDP(311–360) and its mutants.

We next investigated structural transformation of the mutant peptides of TDP(311–360) during the aggregation processes as detected by circular dichroic (CD) spectroscopy (Fig. 4). Previously, wild-type TDP(311–360) showed a negative peak at 206 nm and a shoulder at ~220 nm, and it underwent a transformation from α-helix to a β-sheet-rich conformation and possibly to an amorphous form during aggregation^25^. Similarly, the M337V mutant also showed a shoulder at ~220 nm (Fig. 4a), and when aggregation occurred during incubation, the negative peaks decreased gradually. Secondary structure estimation suggested that the M337V mutant has a similar tendency of structural transformation as the wild type (Fig. 4b). Interestingly, the G335D mutant exhibited a shoulder at ~220 nm in the CD spectra, indicating formation of a small fraction of α-helix. When aggregation occurred, the negative peaks decreased dramatically within half an hour (Fig. 4c). By secondary structure estimation, the percentage of α-helix firstly increased and then decreased, whereas the content of the β-sheet firstly decreased and then increased to an extent of ~60% (Fig. 4d). This suggests that the G335D mutant undergoes a structural transformation from α-helix to a β-sheet-rich conformation and forms amorphous aggregates faster than wild-type TDP(311–360) during aggregation. As for the Q343R mutant, it showed a similar CD spectrum to the wild type and the M337V mutant, but its spectrum curves especially the ellipticities around 222 nm remained unchanged during incubation, suggesting that this mutant loses the ability to structural transformation (Fig. 4e). We also measured the solid-state CD spectra of TDP(311–360) and its mutants to compare their secondary structure formation under amyloid conditions (Fig. 4f)^36^. Wild-type TDP(311–360) formed a typical β-sheet-rich structure in solid amyloid form^25^, while the M337V mutant gave rise to a similar spectrum showing a similar β-sheet structure. The G335D mutant showed a strong negative peak at 221 nm, suggesting that this mutant also forms a β-sheet-rich structure in amyloid. However, Q343R seemed to form a small content of α-helical structure in solid state, indicating that this mutant may not form amyloid aggregates or fibrils after incubation. Together, the G335D mutant has a strong propensity to structural transformation from α-helix to a β-sheet-rich structure and this mutation enhances amyloid formation, whereas Q343R is unlikely to form amyloid aggregates due to its intrinsically disrupted structure.

### The G335D and Q343R mutations influence aggregation and cytoplasmic inclusion formation

**Figure 5:**
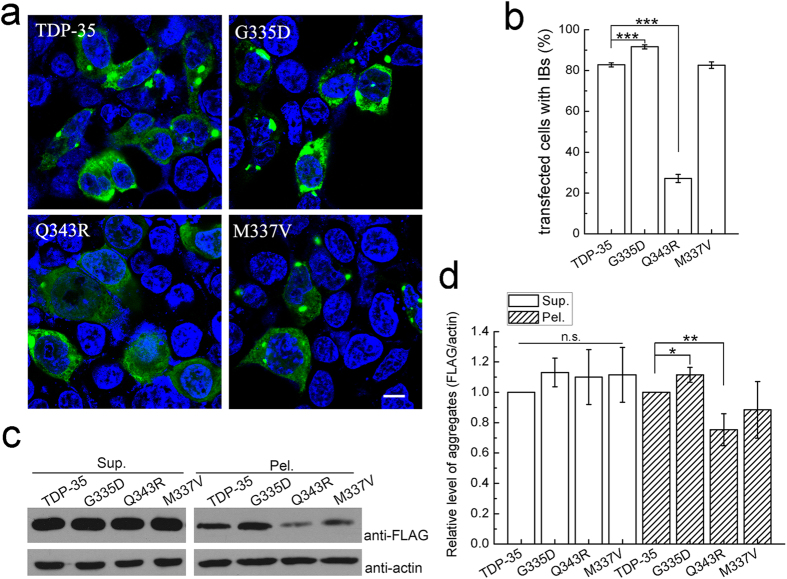
Aggregation and inclusion formation of TDP-35 and its mutants. (**a**) Immunofluorescence microscopic imaging showing inclusion formation of FLAG-TDP-35 and its mutants in HEK 293T cells. Cells were stained with mouse anti-FLAG (green), and the nuclei were stained with Hoechst (blue). Scale bar = 10 μm. (**b**) Quantitation of the cells with inclusion bodies in HEK 293T cells overexpressed with FLAG-TDP-35 and its mutants. Cells were counted and the data were statistically analyzed by one-way ANOVA and shown as Mean ± SD (n = 28–30). ***p < 0.001. (**c**) Supernatant/pellet fractionation assay of the TDP-35 mutants. FLAG-tagged TDP-35 and its mutants were transfected into HEK 293T cells respectively. The cell lysates were fractionated into supernatant (Sup.) and pellet (Pel.) followed by immunoblotting against mouse FLAG and goat actin. (**d**) Quantification of the amounts of overexpressed TDP-35 species in supernatant and pellet fractions respectively. Acitn was set as a control for relative level. Data were normalized, statistically analyzed by one-way ANOVA and represented as Mean ± SD (n = 3). *p < 0.05; **p < 0.01; n.s., no significance.

It has been demonstrated that the C-terminal 35-kDa fragment (TDP-35) of TDP-43 triggers formation of cytoplasmic inclusions^15^, even the inclusions formed by TDP-35 can recruit full-length TDP-43 to form aggregates^37^. To address the importance of these mutations on aggregation and inclusion formation of TDP-35, we visualized the cytoplasmic inclusions of the mutants in cells by confocal microscopy. We constructed FLAG-tagged TDP-35 and its mutants (G335D, Q343R and M337V) and overexpressed them in HEK 293T cells. The imaging showed that, compared with wild-type TDP-35, the G335D mutant, which enhances structural transformation from α-helix to β-sheet, increased the cytoplasmic inclusion formation (Fig. 5a). The percentage of the cells with inclusion bodies for G335D was significantly increased (Fig. 5b). However, the Q343R mutant, which has lost structural transformation, dramatically abrogated the formation of inclusion bodies and only formed punctate foci in cells (Fig. 5a); and the percentage of the cells with inclusion bodies was significantly decreased (Fig. 5b). As a control, the M337V mutation did not affect inclusion formation of TDP-35.

To confirm the above observation, we transfected FLAG-tagged TDP-35 and its mutants respectively into HEK 293T cells, and then performed supernatant/pellet fractionation analysis (Fig. 5c). The data showed that the protein amounts in supernatant remained stable within the experimental errors. However, in the pellet fraction, the amount of G335D aggregates was slightly larger than that of wild-type TDP-35, whereas the pellet fraction of Q343R was significantly decreased (Fig. 5d). As a comparison, the M337V mutant had similar amount of the TDP-35 aggregates with that of the wild type in the pellet fractions.

**Figure 6:**
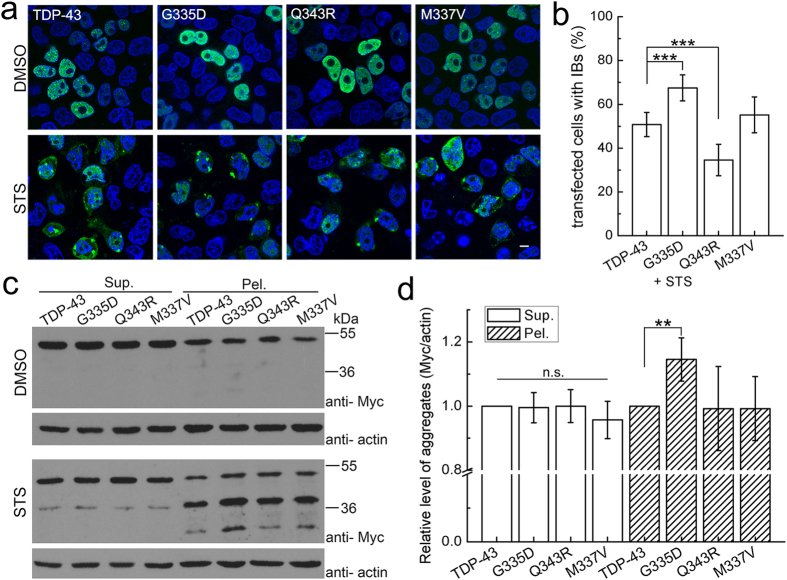
Formation of cytoplasmic inclusions of TDP-43 and its mutants. (**a**) Immunofluorescence microscopic imaging showing inclusion formation of Myc-tagged TDP-43 and its mutants. STS-treated HeLa cells were stained with a C-terminally Myc-tagged antibody for visualizing TDP-43 species (green) and Hoechst for the nuclei (blue). Scale bar = 10 μm. (**b**) Quantitation of the cells with inclusion bodies in STS-treated HeLa cells for Myc-tagged TDP-43 and its mutants. Cells were counted and the data were statistically analyzed by one-way ANOVA and shown as Mean ± SD (n = 15–20). ***p < 0.001. (**c**) Supernatant/pellet fractionation analysis of the deposited species of TDP-43 and its mutants from STS-treated cells. The protein species were detected by Western blotting with an anti-Myc antibody. Sup., Supernatant; Pel., pellet. (**d**) Quantification of the amounts of the proteolytic TDP-35 species from TDP-43 and its mutants. Acitn was set as a control for relative level. Data were normalized, statistically analyzed by one-way ANOVA and represented as Mean ± SD (n = 3). **p < 0.01; n.s., no significance.

As reported previously, TDP-43 is mainly localized in the nucleus that can be extracted into the supernatant. However, when treated with staurospine (STS), TDP-43 is degraded to fragments that cause formation of the cytoplasmic inclusions and deposit in the pellet^15^. To investigate the possible effect of the mutations on inclusion formation, we also transfected C-terminally Myc-tagged TDP-43 and its mutants in HeLa cells. In the cells treated with STS, cytoplasmic inclusions were formed in all TDP-43 species but with different extents (Fig. 6a). For the G335D mutant, the percentage of the cells with inclusion bodies was significantly increased as compared with the wild type, whereas that for Q343R was decreased (Fig. 6b). The aggregation abilities for these TDP-43 mutants were also confirmed by supernatant/pellet fractionation assay on the lysates of STS-treated cells (Fig. 6c). The data showed that the proteolytic fragment TDP-35 generated from wild-type TDP-43 was mostly deposited in the pellet fraction, while the amount of TDP-35 aggregates from the G335D mutant was significantly increased (Fig. 6d). However, the amount of TDP-35 aggregates generated either from Q343R or M337V was similar to that from wide-type TDP-43. Collectively, these results clearly indicate that the G335D mutation enhances aggregation and inclusion formation of TDP-35, whereas Q343R gives an opposite effect.

### Effects of the G335D and Q343R mutations on RNA processing of TDP-43

**Figure 7:**
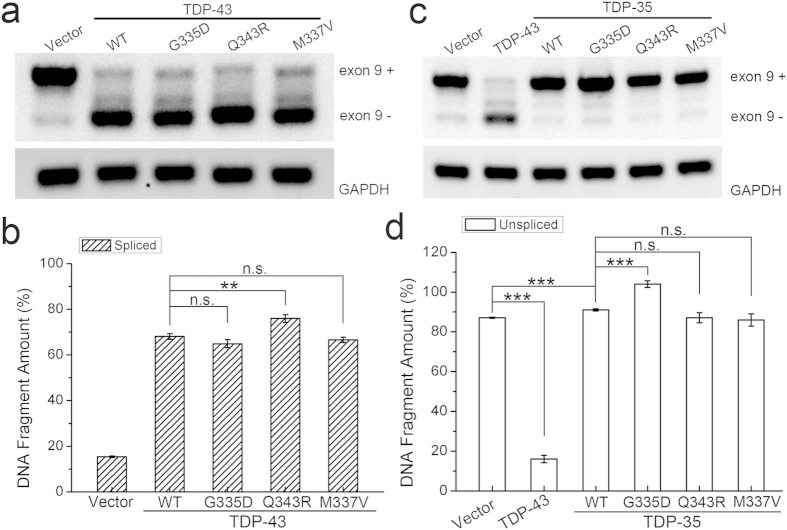
Effects of the mutations on the RNA splicing efficiencies of TDP-43 and TDP-35. (**a**) Effects of TDP-43 and its mutants on CFTR exon 9 splicing. The CFTR splicing reporter construct GT13T5 was transfected into HEK 293T cells with the indicated FLAG-tagged constructs. Exon 9 + denotes unspliced cDNA fragment, while exon 9- represents spliced cDNA. (**b**) Quantification of the spliced RT-PCR products of TDP-43 and its mutants. The total intensity of exon 9 + and exon 9- of vector was set as a control. The intensities of band exon 9- of FLAG-TDP-43 and its mutants were compared with the control, respectively. Data were analyzed by one-way ANOVA and represented as Mean ± SD (n = 3). **p < 0.01; n.s., no significance. (**c**) Effects of TDP-35 and its mutants on CFTR exon 9 splicing. (**d**) As in (**b**) quantification of the unspliced RT-PCR products of TDP-35 and its mutants. ***p < 0.001; n.s., no significance.

As previously reported, TDP-43 can promote CFTR exon 9 alternative splicing and TDP-35 loses the function of RNA processing^8^,^11^,^15^. To ask whether these mutations affect the splicing function of TDP-43 or TDP-35, we co-transfected the CFTR splicing reporter construct GT13T5 and TDP-43/TDP-35 or its mutants into HEK 293T cells and detected RNA splicing efficiencies (Supplementary Fig. S1). As a result, overexpression of TDP-43 strongly promoted CFTR exon 9 exclusion as compared with the mock vector (Fig. 7a). The splicing pattern of the cells transfected with G335D or M337V was somehow similar to that with wild-type TDP-43. However, transfection of Q343R enhanced CFTR exon 9 exclusion more efficiently. It suggests that the Q343R mutant can considerably modify the function of TDP-43 in alternative splicing (Fig. 7b).

As known, TDP-35 may act as an antagonist against endogenous TDP-43 in RNA processing by a dominant-negative mechanism^20^. Our data showed that wild-type TDP-35 could inhibit exon 9 exclusion of endogenous TDP-43 (Fig. 7c, third lane)^15^. Interestingly, when transfection of the G335D mutant in TDP-35 (Supplementary Fig. S1b), the amount of the unspliced band (exon 9+) was significantly increased (Fig. 7c, forth lane), indicating that G335D inhibits alternative splicing more effectively than wild-type TDP-35 (Fig. 7d). This is presumably because aggregation of the G335D mutant in TDP-35 promotes sequestration of endogenous TDP-43 into cytoplasmic inclusions and consequently causes loss-of-function of TDP-43. On the other hand, transfection of Q343R or M337V in TDP-35 did not alter the splicing pattern of endogenous TDP-43. Taken together, these data demonstrate that the Q343R mutant in TDP-43 enhances the splicing efficiency of TDP-43 by increasing the amount of functional TDP-43, whereas G335D in TDP-35 inhibits the splicing by decreasing the amount of endogenous functional TDP-43.

## Discussion

There are many pathologically identified mutations in the amyloidogenic core of TDP-43^7^. The A315T mutation can enhance TDP-43 aggregation and impair axonal mRNA transportation^24^, while Q331K and M337V may increase the amounts of TDP-43 aggregates and cause neuronal dysfunction^33^,^38^,^39^,^40^. Some studies on the Q343R mutation imply that it promotes TDP-43 aggregation and increases the size of neuronal TDP-43 granules in hippocampal neurons^32^,^39^,^41^, but G335D has been identified only in a sporadic Italian ALS patient^7^,^42^.

Structurally, the amyloidogenic core region of TDP-43 forms a helix-turn-helix structure in solution and it is prone to transformation into a hairpin-like β-sheet^25^. We have defined that the G335D mutation in the amyloidogenic core promotes TDP-43/TDP-35 aggregation and cytoplasmic inclusion formation, whereas Q343R gives the opposite action. The structure of G335D has a longer and more flexible loop instead of a relatively rigid turn in the linker region between the two α-helices, which might be beneficial to structural transformation and amyloid aggregation. On the other hand, Q343R has its second helix lost and it may also lose the ability to form a hairpin-like β-sheet structure, which may retard its α-to-β transition and amyloid aggregation. In this aspect, the structural transformation propensity of the amyloidogenic core of TDP-43 is the main determinant for its aggregation and inclusion formation.

TDP-43 is a nucleic-acid binding protein with RNA processing function in nucleus. Mutation, fragmentation and mislocation will alter RNA splicing and cause neurodegeneration^4^,^5^,^15^. We have revealed that two mutations give rise to opposite effects on RNA splicing efficiency, which may result from their altered aggregation properties. Q343R is able to increase RNA splicing efficiency of TDP-43, because its ability to form aggregates or inclusions is decreased. However, G335D in TDP-35 exaggerates the antagonist effect on RNA splicing by endogenous TDP-43 due to its tendency of aggregation and inclusion formation. The altered RNA splicing of TDP-43 caused by G335D or Q343R mutation may further impair a wealth of gene functions in cells, which is probably relevant with the TDP-43 proteinopathies.

Although cytoplasmic inclusions of wild-type TDP-43 are presented in 90% of ALS and 50% of FTLD patients^43^,^44^, mutant forms of TDP-43 can also drive approximately 5% of familial ALS and 4% of sporadic patients^43^,^45^. So far, several animal models studying wild-type or mutant TDP-43 have been established^31^,^38^,^46^,^47^, but the molecular mechanisms by which the disease-associated TDP-43 mutations lead to neurotoxicity in ALS or FTLD are still unclear. Since the G335D and Q343R mutants have been identified to influence the aggregation and inclusion formation of TDP-43 as well as TDP-35 and are implicated in the related disease pathologies, detailed phenotypic analyses in animal models will be essential for elucidating the biological and pathological properties of these TDP-43 mutants.

## Methods

### Construction of plasmids

The cDNAs of human TDP-43 and its fragments were digested and ligated into a pET22b-GFP plasmid by using *Nde* I/*Bam*H I cloning sites to make the GFP-fusion constructs. The cDNA of TDP(311–360) was subcloned into pET32M to get a thioredoxin (Trx)-fused protein, and it was also cloned into pHGB vector to generate GB1-TDP(311–360) with an N-terminal His tag. The TDP-43 and TDP-35 cDNAs were constructed into pcDNA3.1-Myc/His and pcDNA3.1/ FLAG vectors (Invitrogen) respectively for eukaryotic expression. All mutants (Q331K, G335D, M337V and Q343R) were generated by PCR site-directed mutagenesis. All constructs were verified by DNA sequencing.

### GFP-fusion method for assaying the aggregation abilities

The method for assaying the aggregation abilities of GFP-fused TDP-43 fragments and their respective mutants were performed as described previously^25^. Data shown were in triplicate and statistics.

### Protein expression and purification

The Trx and GB1 fused proteins were expressed and purified as described previously^48^. The Trx and GB1 fused proteins were purified through Ni^2+^-NTA following an FPLC Superdex-75 column (GE Biosciences) respectively, and their concentrations were determined spectrophotometrically by using each extinction coefficients.

### Time course of aggregation *in vitro*

The time course of the aggregation process was monitored by ThT fluorescence^35^. Thx fused TDP(311–360) protein and its mutants were diluted to 100 μM with buffer A (25 mM Tris-HCl, 150 mM NaCl, pH8.0). Each 500-μL sample was added with 5 μL of thrombin and incubated at 37 °C with shaking. The purified TDP(311–360) peptide and its mutants were diluted to 100 μM with buffer B (100 mM phosphate, 100 mM NaCl, pH7.0) followed by incubation at 37 °C with shaking. Then each 20 μL of the incubated protein or peptide was added to 980 μL solution with 5 μM ThT in buffer C (50 mM glycine-NaOH, pH 9.0), and the emission intensities at 482 nm were recorded.

### Circular dichroic spectroscopies

All Far-UV CD spectra were recorded on a JASCO J-715 spectropolarimeter (JASCO) at room temperature as described previously^49^. The spectra of the solution samples were acquired scanning from 250 to 190 nm using a 1-mm path-length cuvette, a speed of 10 nm /min, and a time constant of 0.125 s. Each spectrum was processed by three scans of a sample. Data were further processed for noise reduction, base-line subtraction, and signal averaging when needed. The concentration of each protein for time-course incubation is 100 μM in buffer B (100 mM phosphate, 100 mM NaCl, pH7.0). For solution CD measurements, the samples were diluted to a concentration of ~0.25 mg/mL with deionized water, and the data were presented as mean residual molar ellipticities (deg cm^2^/dmol). For the solid-state CD measurements, about 150 μL of the protein solution (ca. 0.50 mg/mL) was cast onto a 2-cm diameter cylindrical quartz glass for evaporating overnight at room temperature, and then the CD spectrum was recorded. The solid-state spectra were presented as ellipticities (mdeg). The secondary structure contents were calculated by using the Model JWSSE-J700 program based on data deconvolution algorithm^50^.

### NMR spectroscopies and structure calculation

NMR data acquirement and processing, and structure calculation were performed as reported previously^25^,^51^. The GB1-fused peptides were applied to enhance peptide solubility and obtain high-quality spectra^52^,^53^. The ^15^N/^13^C-labeled GB1-TDP(311–360) mutant (G335D or Q343R) was dissolved in 20 mM phosphate (pH 6.5), 50 mM NaCl and 8% D_2_O for NMR data acquirements. All NMR spectra were recorded at 25 °C on a Bruker Avance 600-MHz spectrometer equipped with a TCI CryoProbe (Bruker Biospin). The backbone and side-chain chemical-shift assignments were obtained from the spectra of HNCO, HNHA, HNCACB, CBCA(CO)NH, CC(CO)NH, and HCCH-TOCSY. NOE restraints for structure calculations were obtained from ^15^N- and ^13^C-edited NOESY spectra. The NMR data were processed by using NMRPipe and analyzed with SPARKY. The backbone dihedral restraints were derived from TALOS program. The structures were calculated using ARIA2.0 and CNS program, assessed by PROCHECK and displayed by MOLMOL. The structural calculation was performed for 9 cycles and a total of 200 structures were finally obtained. Ten lowest-energy structures were selected and displayed.

### Immunocytochemistry and immunofluorescence microscopy

Cell culture, transfection, Western blotting, immunocytochemistry and confocal microscopy were carried out as described previously^15^. Plasmids were transfected into HEK 293T or HeLa cells with FuGENE HD reagent (Promega) following the manufacturer’s instructions, and then the cells were harvested at 48 h post-transfection. For immunofluorescence microscopy, HEK 293T cells seeded on glass coverslips were grown for 48 h after transfected with FLAG-tagged TDP-35 and its mutants. HeLa cells were grown on glass coverslips for 44 h after transfected with Myc-tagged TDP-43 and its mutants, and then treated with 5 μM staurosporine (STS, dissolved in DMSO) for 4 h. The images were obtained on a Leica TCS SP4 confocal microscope (Leica Microsystems). Mouse monoclonal anti-FLAG (Sigma) or anti-Myc (Cell Signaling) antibody, FITC-conjugated anti-mouse antibody (Jackson Immuno-Research) and Hoechst 33258 (Sigma) were used in these experiments.

### Supernatant/pellet fractionation

The transfected cells were lysed in 100 μL of a RIPA buffer (50 mM Tris-HCl, pH 7.5, 150 mM NaCl, 1 mM EDTA, 1% NP-40, cocktail protease inhibitor (Roche Applied Science)) on ice for 30 min and centrifuged at 15,000 g for 15 min. The supernatant was added with 100 μL of the loading buffer (2% SDS), while the pellet was sufficiently washed with the RIPA buffer for three times and then added with 50 μL of the loading buffer (4% SDS). Equal volume of supernatant and pellet fractions was subjected to SDS-PAGE with 12% acrylamide gel and transferred onto PVDF membranes (PerkinElmer). The mouse monoclonal antibodies against FLAG or Myc, goat anti-actin (Santa Cruz), goat anti-mouse IgG-HRP antibody and rabbit anti-goat IgG-HRP antibody (Jackson Immuno-Research) were used. The proteins were detected by an ECL detection kit (Thermo scientific). The band intensities were quantified by Scion Image software (Scion Corp).

### CFTR exon 9 splicing assay

The method for assaying the CFTR exon 9 splicing was same as the literatures^15^,^54^,^55^. The CFTR splicing reporter TG13T5 minigene was co-transfected with TDP-43/TDP-35 or its mutants respectively in HEK 293T cells. After 48 h post-transfection, the cells were harvested. One half of the cells was subjected to Western blotting to examine the protein expression level, another was applied to measure the CFTR exon 9 splicing/skipping efficiency. The total RNA was extracted with TRIzol reagent (Invitrogen). Mouse monoclonal anti-FLAG (Sigma) and anti-TDP-43 (Abnova) antibodies were used in this experiment. The cDNA was generated by retro-transcription using a ReverTra Ace-α kit (TOYOBO). The secondary PCR was performed using the specific primers according to the literature. GAPDH was used as an internal control. The agarose gel (2%) electrophoresis was carried out, and the intensity of each band was quantified by Scion Image software (Scion Corp).

## Additional Information

**Accession codes**: The coordinates and structure factors have been deposited in the Protein Data Bank with accession codes 2N3X (TDP(311–360)), 2N4G (G335D) and 2N4H (Q343R), respectively. 

**How to cite this article**: Jiang, L.-L. *et al*. Two mutations G335D and Q343R within the amyloidogenic core region of TDP-43 influence its aggregation and inclusion formation. *Sci. Rep.* **6**, 23928; doi: 10.1038/srep23928 (2016).

## Supplementary Material

Supplementary Information
