# Supplementary material for: Two mutations G335D and Q343R within the amyloidogenic core region of TDP-43 influence its aggregation and inclusion formation
Source: Sci Rep. 2016 Mar 31;6:23928. doi: 10.1038/srep23928 (PMC4814915; doi:10.1038/srep23928)
Supplement: Supplementary Information [file srep23928-s1.pdf]

Title:

Two mutations G335D and Q343R within the amyloidogenic core region of TDP-43 influence its aggregation and inclusion formation

Lei-Lei Jiang [1], Jian Zhao [1], Xiao-Fang Yin [1], Wen-Tian He [1], Hui Yang [1], Mei-Xia Che [1] and Hong-Yu Hu [1]

[1] State Key Laboratory of Molecular Biology, Institute of Biochemistry and Cell Biology, Shanghai Institutes for Biological Sciences, Chinese Academy of Sciences. 320 Yue-Yang Road, Shanghai 200031, China.

Correspondence and requests for materials should be addressed to H.-Y H. (email: hyhu@sibcb.ac.cn).

Table S1. Experimental restraints and structural statistics for the G335D and Q343R mutants of TDP(311-360).

Number of experimental restraints	G335D		Q343R
Total unambiguous distance restraints	74		71
Intra residual	40		61
Sequential (| i – j | = 1)	18		7
Medium range (2 ≤| i – j | ≤ 4)	16		3
Long range (| i – j | ≥ 5)	0		0
Dihedral angle restraints			
φ:	21		33
ψ:	21		33
Structure model statistics			
RMSD from experimental restraints			
NOE distances（Å）	0.024+/-0.005		0.037+/-0.000
Dihedral angles (deg.)	0.83/-0.19		0.66+/-0.09
RMSD from idealized geometry			
Bonds（Å）	0.003+/-0.000		0.003+/-0.000
Angles (deg.）	0.432+/-0.032		0.405+/-0.016
Impropers (deg.）	1.24+/-0.174		1.22+/-0.158
Ramachandran analysis			
Residues in most favored regions (%)	70.2		84.4
Residues in additionally allowed regions (%)	24.8		12
Residues in generously allowed regions (%)	2.3		1.38
Residues in disallowed regions (%)	2.67		2.25
Average atomic RMSDs			
All residues			
Backbone atoms（Å）	9.16+/-1.87		7.29+/-1.38
Heavy atoms（Å）	9.94+/-1.88		8.49+/-1.38
Secondary structures	(321-330)	(339-343)	（322-333）
Backbone atoms（Å）	0.50+/-0.18	0.24+/-0.09	0.78+/-0.21
Heavy atoms（Å）	1.32+/-0.18	1.18+/-0.23	1.60+/-0.28

Figure S1

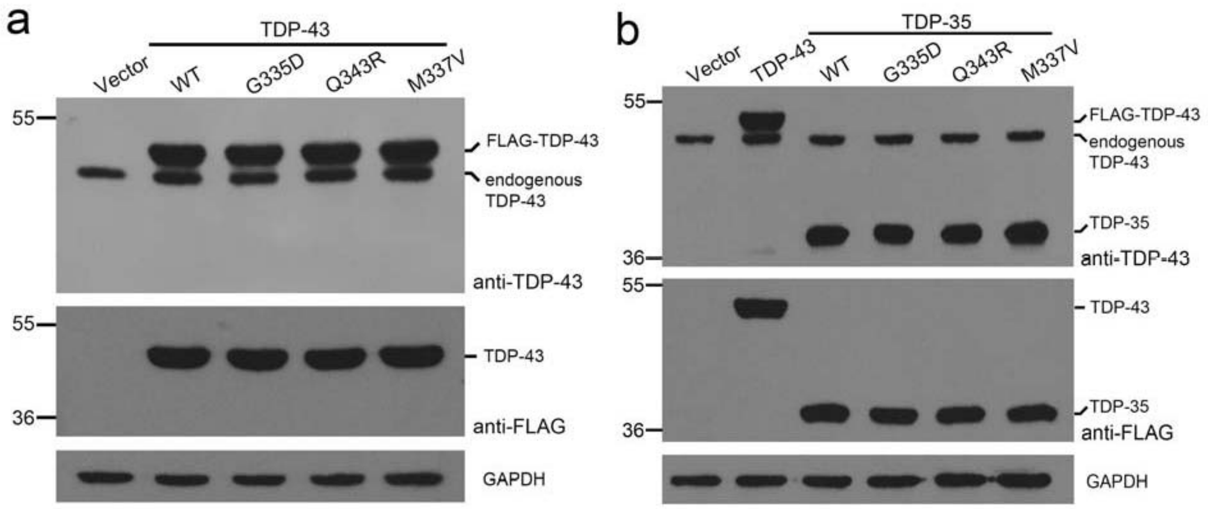

Figure S1 │ Expression levels of the protein species for CFTR splicing assay. (a) Western blotting analysis using the anti-TDP-43 and anti-FLAG antibodies shows that the expression products of both endogenous and FLAG-tagged TDP-43 were at similar levels. (b) Western blotting analysis shows that the expression products of both endogenous TDP-43 and FLAG-tagged TDP-35 were at similar levels.
